# Quantitative Proteomic Profile of Psoriatic Epidermis Identifies OAS2 as a Novel Biomarker for Disease Activity

**DOI:** 10.3389/fimmu.2020.01432

**Published:** 2020-07-31

**Authors:** Yuan Zhou, Ping Wang, Bing-Xi Yan, Xue-Yan Chen, Lilla Landeck, Zhao-Yuan Wang, Xin-Xin Li, Jing Zhang, Min Zheng, Xiao-Yong Man

**Affiliations:** ^1^Department of Dermatology, Second Affiliated Hospital, Zhejiang University School of Medicine, Hangzhou, China; ^2^Ernst von Bergmann General Hospital, Teaching Hospital of Charité, University Medicine Berlin, Humboldt University Berlin, Potsdam, Germany

**Keywords:** psoriasis, proteomics, epidermis, iTRAQ, OAS2

## Abstract

Psoriasis is a common chronic inflammatory systemic disease. Epidermal proteins are considered to be important in maintaining skin barrier function, innate immunity, and inflammation. To define more possible roles of the epidermis in the pathogenesis of psoriasis, quantified proteomic analysis was used to screen and analyze the differentially expressed epidermal proteins between 16 psoriasis patients and 15 healthy controls. Upregulated differential expression proteins (DEPs) include several significant functional protein clusters, including antimicrobial peptides (AMPs) and antiviral proteins (AVPs). The levels of 2–5-oligoadenylate synthase 2 (OAS2) in both epidermis and serum levels were significantly elevated in psoriasis and were also positively correlated with Psoriasis Area Severity Index (PASI) scores and Body Surface Area (BSA) scores. Moreover, OAS2 expression in psoriatic skin significantly decreased after IL-17R mono-antibody treatment. It has been clarified that inflamed keratinocytes were the main source of abnormally increased OAS2 in psoriasis skin by immunofluorescence and primary cell cultures. Keratinocyte-derived OAS2 can be induced by not only IFNβ, but also psoriasis associated cytokines like IL-17A and IL-6. This study revealed that AMPs and AVPs are two important functional protein clusters altering innate immune in psoriatic epidermis. OAS2 is a novel potential sensitive biomarker, which could predict the severity and activity of psoriasis, and could also be used as an indicator to evaluate or monitor the efficacy of clinical treatment.

## Introduction

Psoriasis vulgaris is a chronic immune-mediated inflammatory systemic disease, characterized by raised, well-demarcated, erythematous plaques with adherent silvery scales (1). The histopathological features are comprised of epidermal thickening, parakeratosis, elongated rete ridges, angiogenesis, and lymphocytic infiltration (1, 2). Approximately 2% of the world's population are affected by psoriasis (3). To date, it is still debatable whether the epidermal cells or the dermal immune cells play a more important role in the pathogenesis of psoriasis.

High-throughput profiles including genome, epigenome, transcriptome, proteome, and metabolome have been performed in the last two decades (4–18). These omics-based discoveries have identified many promising biomarkers. However, genome or transcriptome datasets cannot predict precise functional proteins altered in diseases (19). There is a shift between differential expression genes (DEGs) and differential expression proteins (DEPs) in the same human psoriasis sample through transcriptome-proteome integration analysis (13).

Since 2005, proteomics has been employed in psoriatic research by more than 20 research groups (4, 6, 8, 11, 13, 15–17, 20). Previous studies have detected novel biomarkers which could monitor the efficacy or toxicity of treatment (21). However, these studies used a variety of different specimens (e.g., serum/plasma, saliva, skin biopsies; cultured, or sorted cells) and applied diverse methodologies for their investigations, which makes the results different and incomparable. To our knowledge, only two studies have focused on the epidermal proteomics for psoriasis so far. One study compared lesional and non-lesional epidermis digested from the skin of psoriasis patients by trypsin (6), whereas another group analyzed the skin tissue taken by keratome that included epidermis and upper dermis (8). The detected samples in those two studies were not precise psoriatic epidermis.

To understand the contribution of the epidermis in psoriasis, we aimed to identify alterations of epidermal proteins with a precise source. To this end, the epidermis was separated from 16 lesional skin samples of psoriasis patients and 15 normal skin samples of healthy controls by dispase digestion. Quantitative proteomics analysis by iTRAQ labeled LC-MS/MS revealed that proteins within psoriatic lesional epidermis significantly changed. Moreover, 2–5-oligoadenylate synthase 2 (OAS2) was defined as a potential biomarker for the severity and activity of psoriasis.

## Materials and Methods

### Skin and Serum Samples

Skin biopsies were obtained from 16 patients suffering from psoriasis vulgaris (gender, age, BSA, PASI, and sample taken site details shown in Supplementary Table 1). To avoid treatment interference, skin biopsies were taken from patients who did not perform any topical treatments for at least 2 weeks, and any systemic immunosuppressive medications and phototherapy for at least 4 weeks. Fifteen healthy subjects (gender and age details shown in Supplementary Table 2) were used in our study.

Serum samples were obtained from 32 patients with psoriasis vulgaris (24 males and eight females, PASI range 0.6–66.8, mean 17.38; BSA range 1–90%, mean 24.03) and 26 healthy subjects (18 males and eight females). The study was approved by the Ethics Committee of the Second Affiliated Hospital, Zhejiang University School of Medicine. Every patient and donor agreed to participate in this study and signed the Informed Consent of Biopsy.

### Tissue Processing and Protein Extraction

The epidermis was separated after 0.5% dispase (GibcoTM) digestion at 4°C overnight.

The samples were individually milled to powder in a mortar cooled with liquid nitrogen. The powder was mixed with lysis buffer and centrifuged, and the supernatant was transferred to a clean tube. Each sample was digested with Trypsin Gold (Promega, Madison, WI, USA) for peptide preparation at 37°C for 16 h, then desalted with C18 cartridge to remove the high urea. Finally, the desalted peptides were dried by vacuum centrifugation.

### Isobaric Tags for Relative and Absolute Quantitation Labeled Liquid Chromatograph-Mass Spectrometer (iTRAQ-Labeled LC-MS/MS)

The desalted peptides were labeled with iTRAQ reagents (iTRAQ® Reagent-8PLEX Multiplex Kit, AB Sciex), following the manufacturer's instructions (AB Sciex, Foster City, CA). The tryptic peptides were monitored at UV 214 nm. Eluent was collected every minute and then merged to 15 fractions.

Fractions from the first dimension RPLC were dissolved with loading buffer and then separated by a C18 column. Q-Exactive HF-X mass spectrometer was operated in positive polarity mode with capillary temperature of 320°C.

### The Enzyme-Linked Immunosorbent Assay (ELISA)

Serum samples were incubated in a pre-coated human OAS2 ELISA plate (LSBio) for 1 h at 37°C, then the detect antibody was added and incubated for 1 h at 37°C. The plate was washed three times followed by an incubation with Streptavidin-HRP Complex for 30 min at 37°C. After washing five times, we added TMB substrate, then terminated with stop solution afterwards. The optical density (OD value) was detected immediately using a reader at 450 nm (BioTek, ELx808).

### Primary Human Epidermal Keratinocyte and Fibroblast Culture

Primary psoriatic lesional epidermal keratinocyte (PLEK), psoriatic non-lesional epidermal keratinocyte (NLEK), and dermal fibroblasts were cultured according to our previous work (22–24). Briefly, skin biopsy samples were first incubated with 0.5% dispase at 4°C overnight. Then, the epidermis was peeled off from the dermis and incubated in 0.25% (w/v) trypsin for 10 min, neutralized by 10% FBS and centrifuged at 1,000 rpm. Keratinocytes were cultured in a humidified atmosphere with 5% CO_2_ at 37°C in serum-free medium from Millipore. The dermis was incubated with 1 mg/ml collagenase (Sigma) at 37°C for 90 min and then cultured in 10% FBS DMEM. The cells were grown until 65% confluence.

### HE Staining and Immunofluorescence (IF)

The separated epidermis was embedded in OCT and sectioned by freezing microtome (Leica). The sections were stained with hematoxylin and eosin. Images were captured by NDP view software after being scanned by a Nano Zoomer (Hamamatsu).

For IF staining of OAS2, whole skin samples embedded by OCT were sectioned. Slices were blocked with mouse serum (Vector Lab) and incubated with monoclonal OAS2 antibody (Santa Cruz) followed by incubation with Alexa Fluro 488-conjugated secondary antibody (Invitrogen). For each case, a negative control incubated with non-immune mouse IgG (Sigma-Aldrich) was included. IF assay was pictured by fluorescence microscope (Leica DM5500B).

### Western Blot Analysis

Cells were lysed with RIPA buffer and lysates were boiled with 5 × Loading buffer for 10 min. The samples were then separated with SDS-PAGE gel and immunoblotted with indicated antibodies, followed by an incubation with a secondary antibody. The immunoreactive bands were detected using ECL Substrate (Thermo Scientific™).

### Imiquimod (IMQ) Induced Psoriasis-Like Mouse Model

Female C57BL/6 mice at 6 weeks old were supplied by SLAC Laboratory Animal Co. (Shanghai, China). The protocol was approved by the Ethics committee of the Second Affiliated Hospital, Zhejiang University School of Medicine. All efforts were made to minimize the suffering of the mice. The mouse was shaved on the dorsal skin. The imiquimod cream with a dose of 62.5 mg was topically applied to the mouse back every day for 6 consecutive days. The sham controls were treated similarly with the vanishing cream base.

### Data and Statistical Analysis

The raw resulting spectra from each fraction were searched separately against uniprot_homo_sapiens_2017.11.3 fasta database by the search engines: Proteome Discoverer (Thermo Scientific). The searched parameters were as follows: A mass tolerance of 10 ppm for precursor ion scans and a mass tolerance of 0.02 Da for the product ion scans were used.

The DEPs were identified by fold change (FC) of quantitative psoriasis vs. healthy control (P/H). The protein was considered as an up-regulated DEP when its FC was higher than 1.500. And, the FC of down-regulated DEP was <0.667 (1/1.5). *P-*values were all less 0.01. The protein quantification results were statistically analyzed using the Mann-Whitney Test.

The publicly available microarray dataset of paired non-lesional, lesional, and brodalumab treated lesional biopsy psoriasis samples (*n* = 99) were from Gene Expression Omnibus (GEO) (GSE53552) (25).

Spearman's rank correlation coefficient was used to analyze the correlation between epidermal OAS2 level and PASI score, BSA.

Pearson correlation coefficient was used to evaluate the correlation between the OAS2 level of serum and PASI score, BSA of patient.

Other statistical analyses were performed with Graphpad Prism v.8.2.1 for Mac (GraphPad Software, CA, USA).

## Results

### Identification of Epidermal Proteins and DEPs in Psoriatic and Normal Epidermis

**Figure 1:**
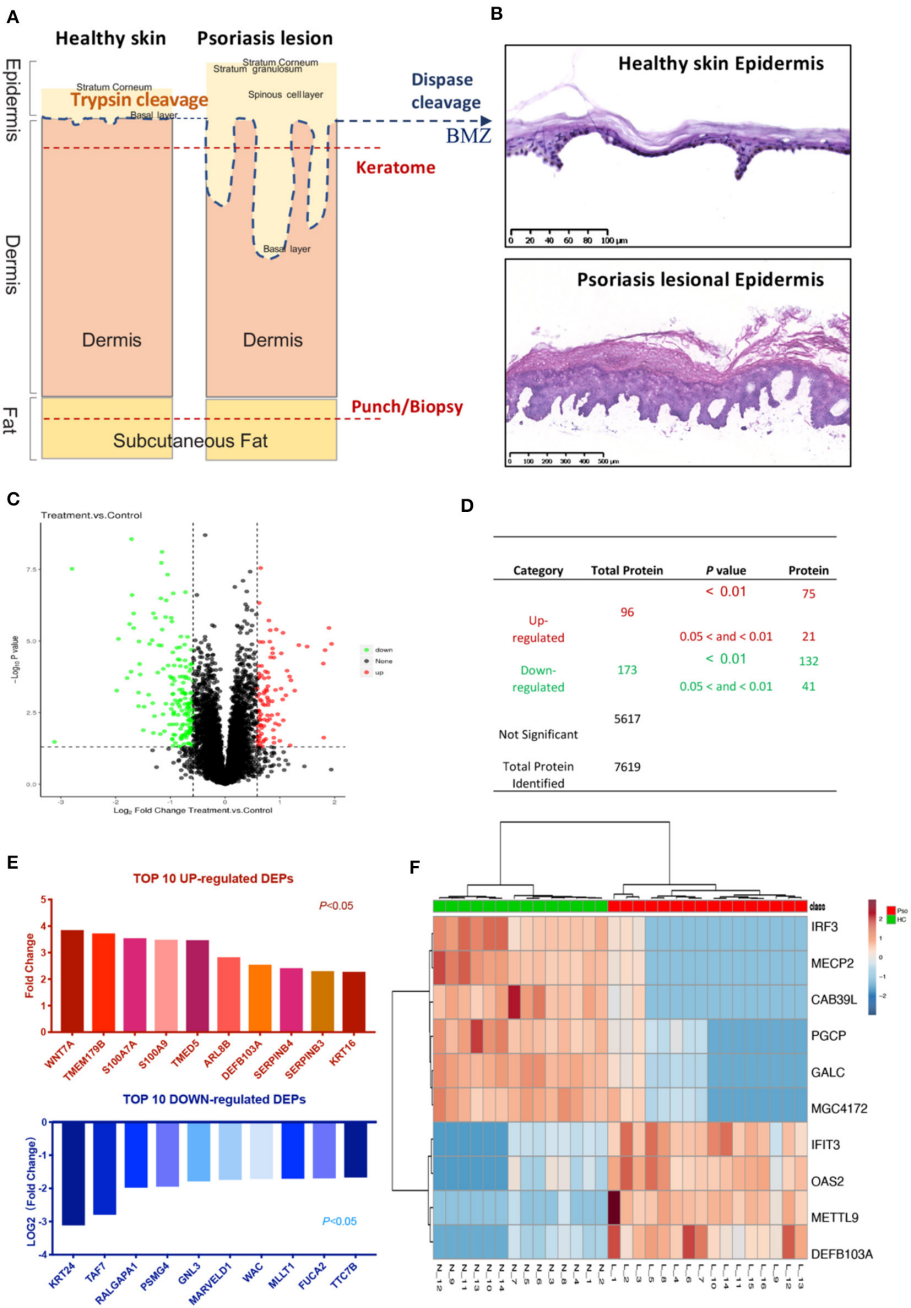
Identification of psoriatic epidermal proteins. **(A)** Tissue taken and processed by different methods. Blue dashed lines (dispase digestion): level of epidermal-dermal junction. Two red dashed lines: typical depths of keratome and punch biopsies. **(B)** HE staining of healthy and psoriatic epidermis after dispase digestion, peeled off from skin. **(C)** Volcano plot: detected proteins by FC and *P-*value. **(D)** List numbers of identified proteins. **(E)** Top 10 up- and down-regulated DEPs ranked by fold change and Log_2_(fold change) (*P* < 0.05). **(F)** Heat map with the most significant 10 DEPs according to the quantitative expression level of sample from the two groups. The DEPs ID (for uniprot database), description, coded gene, fold change and *P*-value were shown in Supplementary Table 3. The encode gene names of proteins were listed under the graph **(E)** and on the right of heatmap **(F)**.

In contrast to previous epidermal proteomics studies, our target-tissue included intact epidermis, along with the epidermal-dermal junction, and basement membrane zone (BMZ) (Figure 1A). To test the integrity of the epidermis after dispase digestion, the separated epidermis was stained with Hematoxylin and Eosin (HE). Both healthy and psoriatic epidermis were completely separated from the skin. Basal cells were round and dissociated from the dermis, but still connected to prickle cells and distributed regularly. There was no obvious change in other epidermal layers, and the psoriatic epidermis was thickened (Figure 1B). A total number of 7,619 proteins and 269 DEPs were identified in the epidermis from healthy subjects and psoriasis patients (*n* = 15/16). In the psoriatic epidermis, we identified 96 up-regulated and 173 down-regulated proteins (Figures 1C,D).

Among all identified proteins based on GO (Gene Ontology) annotation, the main groups of functional proteins were binding proteins (protein binding and ATP binding), antigen processing and presentation proteins, which are most likely to be located in the nucleus and membrane (Supplementary Figure 1A), indicating that the epidermal cells in an active status may participate in innate immune to maintain skin barrier homeostasis. However, the GO analysis found that DEPs were mainly involved in biological processes (BP), such as signal transduction, transport, proteolysis, and small GTPase mediated signal transduction, instead of antigen processing and presentation (Supplementary Figure 1B). Thus, the alteration of BP-associated proteins may reveal the main responsible proteins in psoriatic epidermis.

To assay the different statuses in psoriatic and healthy epidermis, the top 10 up-regulated and down-regulated DEPs ranked by FC from all 269 DEPs (Supplementary Table 3) were listed (Figure 1E). The top up-regulated DEPs included calcium-ion binding and antimicrobial proteins (AMPs) (S100A7, S100A9, β-Defensin 103), Serpins family (Serpin B3 and Serpin B4), Keratin16, and others.

Interestingly, more novel proteins were significantly changed in psoriatic epidermis, like OAS2, Interferon-induced protein with tetratricopeptide repeats 3 (IFIT3), IRF3, MeCP2, and so on (Figure 1F).

### Protein-Protein Interactions (PPI) and Correlation of DEPs Psoriatic Epidermis in Active Status

To determine abnormally activated signaling pathways in psoriatic epidermis, we performed a Kyoto Encyclopedia of Genes and Genomes (KEGG) pathway analysis (Supplementary Figure 2), which found five pathways that are involved in viral infection associated immune response and are listed as map05164 (Influenza A), map05161 (Hepatitis B), map04622 (RIG-I-like receptor signaling pathway), map05160 (Hepatitis C), and map05166 (HTLV-I infection). These pathways were remarkably associated with the following DEPs: OAS2 (up), IFIH1 (up), STAT (up), Mx1 (up), importin-α (up), PABPN1 (down), IRF3 (down), MHC-II (down), and transmembrane protease serine 13 (down). Furthermore, lots of proteins were enriched in metabolism such as sphingolipid and steroid metabolism Supplementary Figure 2).

**Figure 2:**
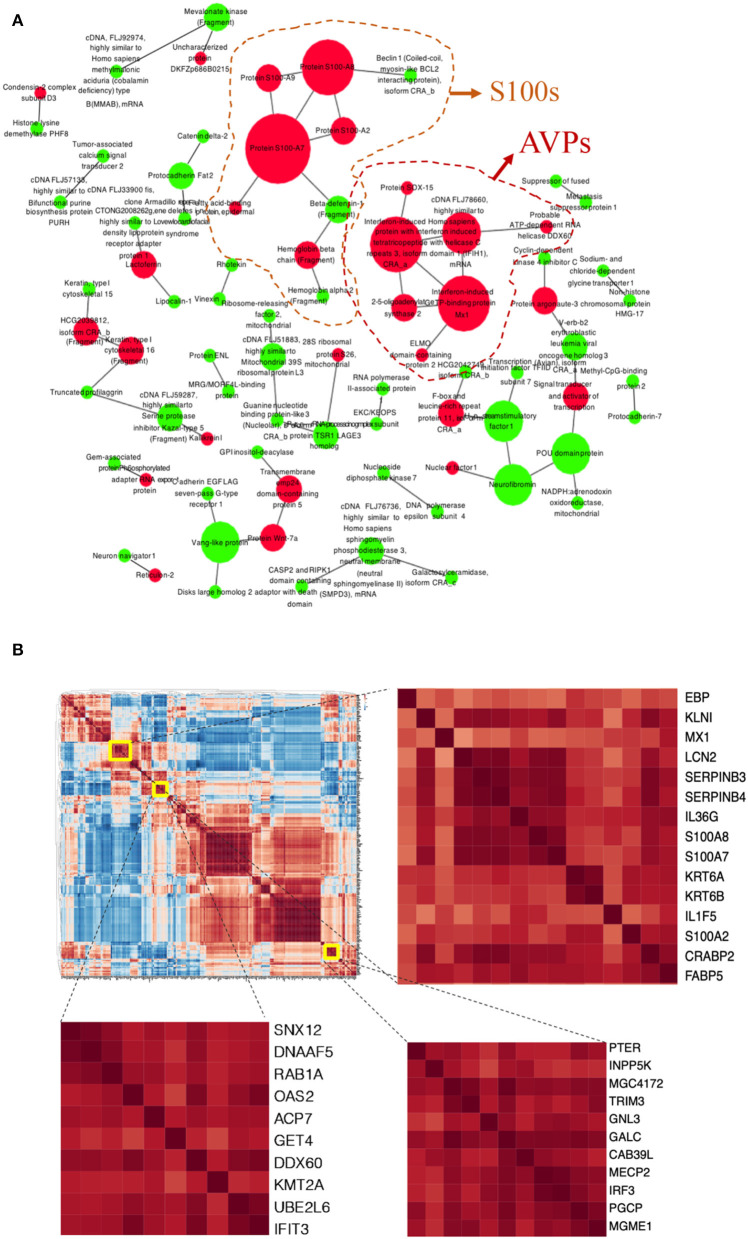
Protein-Protein Interactions (PPI) and correlation in the DEPs of psoriatic epidermis. **(A)** PPI visualized potential interactions among DEPs. Up-(red) and down-(green) regulated DEPs showed. Size of the dot according to the number of proteins it interacted with. **(B)** Correlation heatmap of DEPs, enlarged parts shown at right.

PPI network also highlighted calcium ion-binding proteins, AMPs (S100A7, S100A8, S100A9, S100A2, and defensins), and antiviral immune associated proteins (Mx1, OAS2, interferon-induced proteins) predominated in pathological protein interaction (Figure 2A). These proteins were overactivated in psoriatic epidermis.

To further the interaction of DEPs, a correlation analysis of all DEPs implied that those proteins potentially affected each other (Figure 2B). Mx1, LCN2, Serpin B3, Serpin B4, IL-36γ, S100A8, S100A7, and Kertin 6 were highly correlated, suggesting that this group of proteins may have potential functional interactions in pathological epidermis (Figure 2B).

### OAS2 as a Novel Candidate Biomarker for The Severity and Activity of Psoriasis

**Figure 3:**
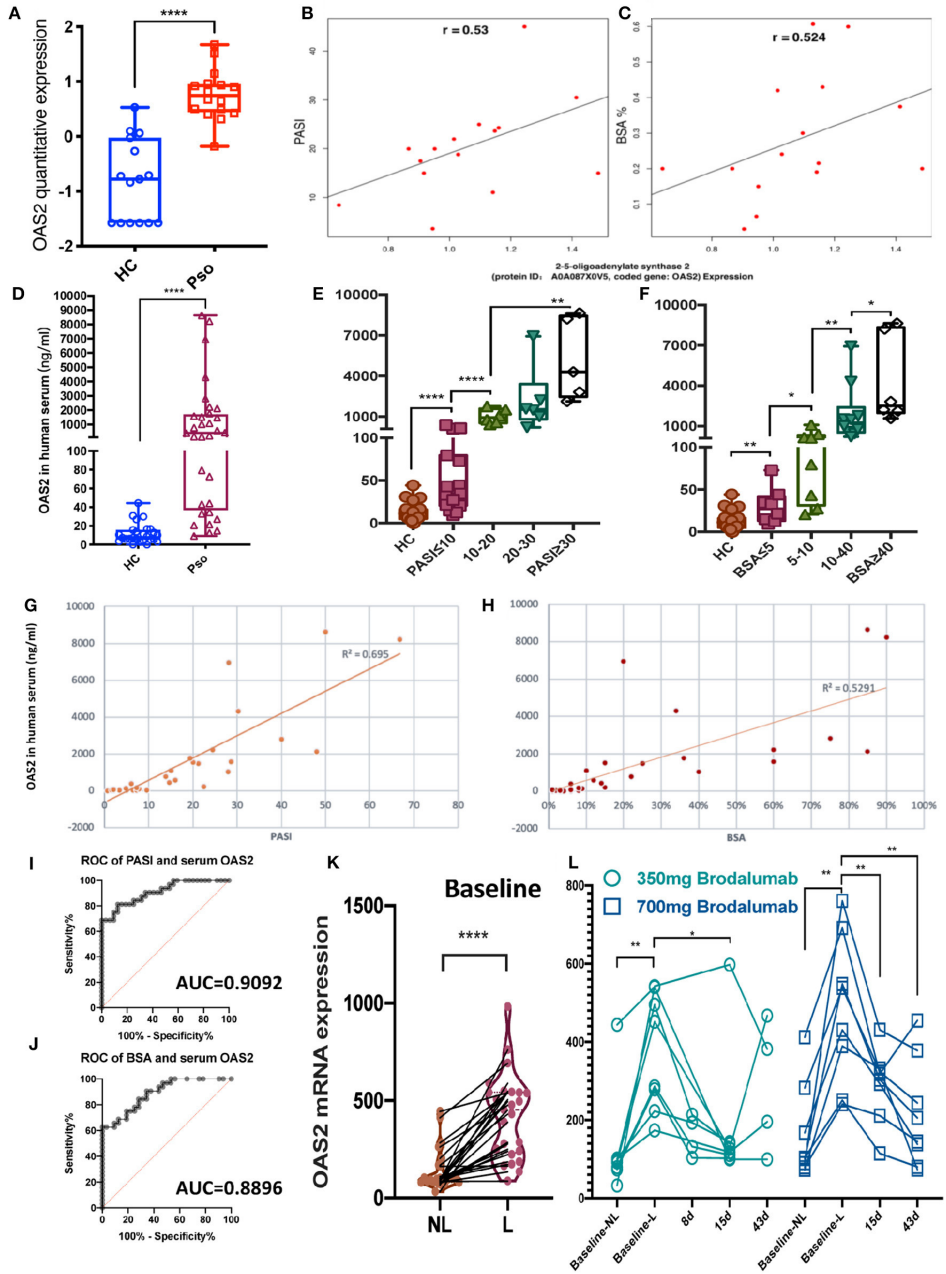
OAS2 as a novel candidate biomarker for the activity of psoriasis. **(A)** Box graph shown epidermal OAS2 level (min to max) in healthy control (HC) and psoriatic (Pso) (*P* = 0.0000181). **(B,C)** Spearman's rank correlation between epidermal OAS2 level and PASI **(B)** (*P* = 0.035), BSA **(C)** (*P* = 0.045), respectively. **(D–F)** Serum OAS2 (ng/ml) level in HC (*n* = 26) and Pso (*n* = 32), and in groups by PASI **(E)** and BSA **(F)** (Mann-Whitney test, *P < 0.05, **P < 0.01, ****P < 0.0001). **(G–J)** Pearson correlation between psoriatic serum OAS2 level (ng/ml) and PASI **(G)**, BSA **(H)**, and ROC of them **(I,J)**, respectively. **(K,L)** OAS2 level analysis of psoriasis skin microarray data between non-lesional skin (NL) and lesional skin (L) **(K)**, between baseline and after brodalumab treatment **(L)**.

To further understand the relationship between DEPs and the severity of psoriasis, the quantitative DEPs were correlated with the psoriasis area and severity index (PASI) and the body surface area (BSA). OAS2 (2–5-oligoadenylate synthase 2, Protein ID: A0A087X0V5) was outstanding in these analyses. OAS2, an enzyme that is coded by the gene OAS2 in humans, was acknowledged as an antiviral protein (AVP) (26). In our study, the expression level of the OAS2 protein was higher in the psoriatic epidermis compared to the controls (FC = 1.82, *P* < 0.0001) (Figure 3A). Interestingly, increased OAS2 was positively correlated with both PASI and BSA scores (Figures 3B,C).

Sampling of serum is easier and safer for patients and more cost-effective in the clinical routine than sampling of skin. To define OAS2 as a potential psoriasis biomarker, the serum level of OAS2 detected by ELISA was significantly higher in psoriasis patients (*n* = 32) than in healthy controls (HC, *n* = 26) (*P* < 0.0001, Figure 3D). More interestingly, even in psoriasis patients with a low PASI score or low BSA, the serum OAS2 level was significantly higher when compared to HC (Figures 3E,F). Remarkably, the OAS2 expression in serum was positively correlated with PASI and BSA (Figures 3G–J, Pearson correlation coefficient *R*^2^ = 0.695 and 0.529, respectively; *P* < 0.01 both; area under the cover (AUC) = 0.9092 and 0.8896, respectively). Therefore, OAS2 may be a potential sensitive marker for psoriasis, which can also reflect the severity of psoriasis.

To assay OAS2 as a potential therapeutic biomarker, we analyzed a public microarray dataset of a dose-response and time-course study on psoriasis treatment by analyzing the transcriptional level of skin biopsy after anti-IL-17R (brodalumab) treatment (25). The expression of OAS2 in lesional skin was dramatically higher than non-lesional skin from the same patients (Figure 3K), and decreased after brodalumab treatment for 8, 15, and 43 days, especially in patients treated with a higher dose (Figure 3L).

### Keratinocyte Derived OAS2 Contributes To Psoriasis

**Figure 4:**
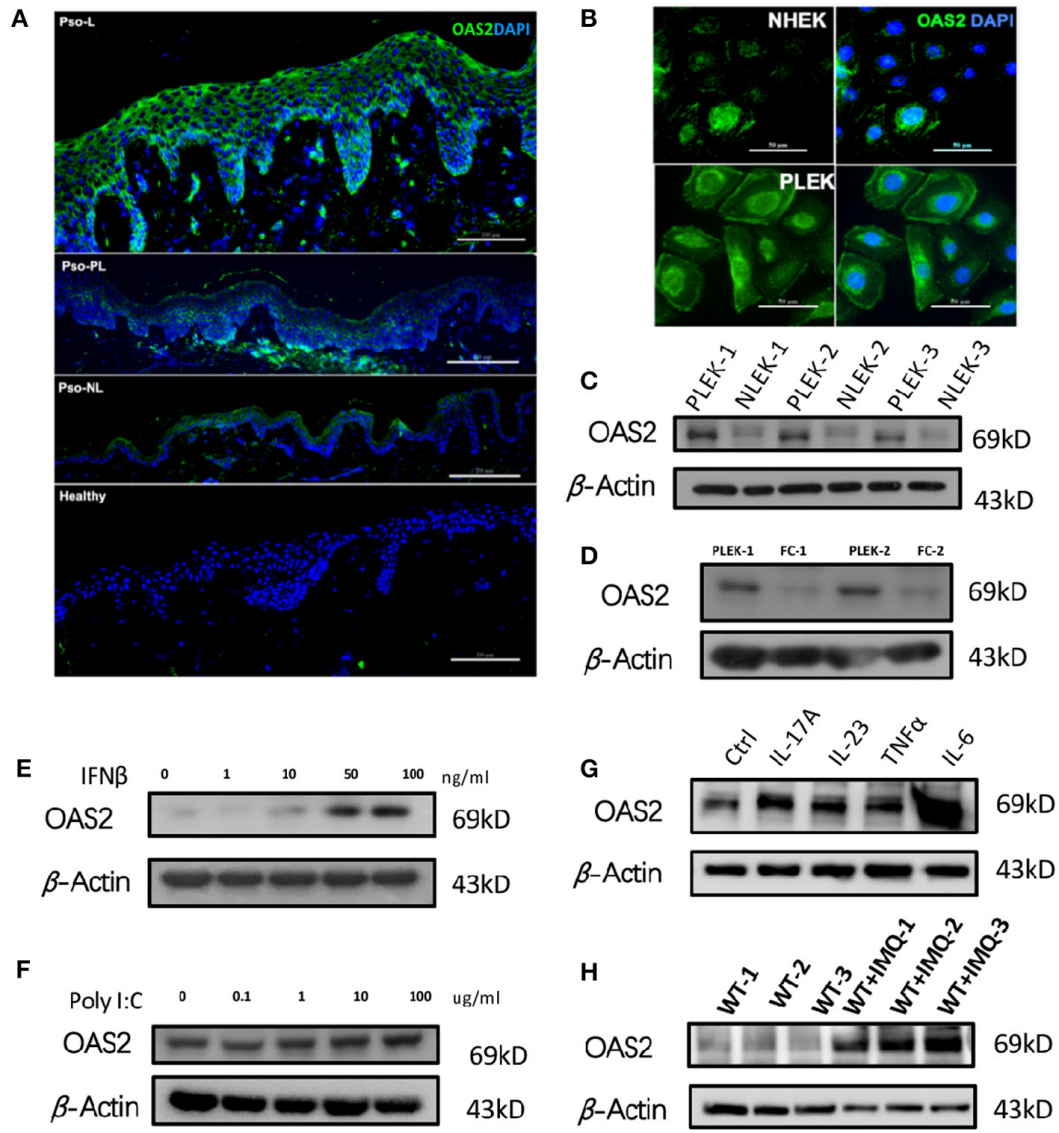
Keratinocyte derived OAS2 contributes psoriasis. **(A,B)** IF analysis of OAS2 in healthy—and psoriatic skin **(A)**, cultured normal human epidermal keratinocytes (NHEK) and psoriatic lesional epidermal keratinocytes (PLEK) **(B)**. **(C–G)** Western Blot analysis of OAS2 in paired PLEK and NLEK (*n* = 3), paired PLEK and fibrocytes (FC) (*n* = 2), after 24 h treatment of IFNβ **(E)**, Poly I:C **(F)**, psoriasis associated cytokines IL-17A (50 ng/ml), IL-23 (50 ng/ml),TNFα (50 ng/ml), and IL-6 (50 ng/ml). **(H)** The OAS2 level in mouse epidermis after imiquimod (IMQ) treatment for 5 days (*n* = 3).

Next, to determine the expression and localization of OAS2, we compared the OAS2 expression in different skin areas from patients suffering from psoriasis and HC. OAS2 was expressed significantly higher in psoriatic-lesional (Pso-L) epidermis when compared with peri-lesional (Pso-PL), non-lesional (Pso-NL) and healthy controls (Figure 4A), and the localization of OAS2 was mainly in keratinocytes, indicating that keratinocytes may be a major source of OAS2 production in psoriasis. We therefore detected the expression of OAS2 in human primary keratinocytes (Figure 4B), and found that OAS2 was more actively expressed in primary psoriasis lesional epidermal keratinocyte (PLEK) than normal human epidermal keratinocyte (NHEK) and psoriasis non-lesional epidermal keratinocyte (NLEK), but rarely expressed in fibroblasts (Figures 4C,D).

Notably, OAS2 can be up-regulated by type I interferon-IFNβ, IL-17A, IL-23, TNFα, and IL-6 in NHEK, but was not changed after poly I:C treatment (Figures 4E–G). Similarly, in the classical imiquimod (IMQ) induced psoriasis-like mouse model, OAS2 expression was significantly increased (Figure 4H). Therefore, in addition to antiviral immune response, OAS2 possibly contributes to the pathogenesis of psoriasis which makes it a candidate biomarker for further research.

## Discussion

As a global health problem, psoriasis affects more than 125 million people worldwide with lifelong torment (3). Even new targeted therapies have a satisfactory clinical outcome, but to date there is still no cure for psoriasis (27). More noteworthy is that novel targeted therapies directly regulate epidermal inflammation and hyperplasia (28). However, epidermal key proteins that are crucial for the pathogenesis of psoriasis are still insufficiently understood. To identify that, our study compared proteins from psoriatic and healthy epidermis at a quantitative proteomics level. Novel potential functional proteins that are associated with an antiviral immune response and metabolism were found in the psoriatic epidermis. A novel protein biomarker, OAS2, was positively associated with the severity and activity of psoriasis.

Proteomics can provide a better quantitative and functionally systemic analysis of disease associated proteins than genomics or transcriptomics. Some of the results from 20 more psoriasis proteomic studies (21) shared similar altered proteins, while most of them were different. These differences may be due to different tissue sources and different processing methods.

Therefore, it is important to select an accurate source of proteins. The best way is to determine the source of DEPs from epidermis and dermis separately. Two previous psoriasis proteome studies aimed to focus on the epidermis separated by keratome and trypsin cleavage, respectively (6, 8). Trypsin treatment used for a dermal–epidermal split would leave numerous basal cells adherent to the dermis in normal skin (29). Furthermore, epidermal psoriasiform hyperplasia with elongation of the rete ridges into the dermis would make trypsin separation, to obtain the whole epidermis, less possible. Only a “clean” separation by dispase, as shown in Figure 1B, guarantees a precise source of detected proteins. It is even more critical that the separated epidermal cells should be alive with a potential proliferation potency for primary culture (22).

It is well-known that uninvolved psoriatic skin is not innocent as a potential “psoriatic milieu.” Therefore, proteomic studies investigating and comparing healthy and psoriatic epidermis may be more promising when searching for key proteins responsible for the transformation from a healthy into an inflamed stage. That is the reason healthy controls were included in this study.

Our data confirmed several proteins already known to be associated with psoriasis, including S100A7, S100A9, beta-defensin 3(DEFB103A), Elafin (PI3), serine protease inhibitors B3, and B4 (Supplementary Table 3) (6, 8). These findings suggest that our data is credible.

Wnt signaling pathway contributes to abnormal proliferation of epidermal cells in psoriasis (30, 31). Wnt-7a was the most significantly up-regulated protein in our analysis (Figure 1E), particularly when compared with S100A8 and S100A9. Hence, Wnt-7a may play a key role in Wnt signaling pathway-primed proliferation of epidermal cells.

Additionally, IL-1 and IL-36γ are highly increased in psoriatic epidermis (Figure 2B and Supplementary Table 3). Therefore, our results highlight the role of the IL-1 family in psoriasis, which is in accordance with many recent studies focusing on the IL-1 family in psoriasis (32, 33).

In addition, our pathway analysis implied that virus associated immune response/innate immunity and lipid metabolism are dysfunctional in psoriatic epidermis. Adheres junction, Hedgehog signaling pathway and Cell cycle pathway (Supplementary Figure 1) were highlighted in our data, indicating that psoriasis epidermal cells are over-proliferated with skin barrier dysfunction, which may be attributed to a group of down-regulated proteins, including vinexin, Smad4, FERM, metastasis suppressor protein 1 and catenin delta-2 as shown in our data.

Furthermore, our GO enrichment analysis showed that five enrichment DEPs related to immune response (GO ID:0006955) are upregulated, including IL-1 (IL1F5), IL-36γ, S100A8, S100A9, and OAS2. In a recent epidermal transcriptomic study that screened CD45neg non-immune cells, the authors found that the genes of OAS2, DEFB4A, PI3, DEFB4B, SERPINB3, KYNU, S100A9, and S100A7A were up-regulated (34). Our dataset confirmed part of these transcriptomic changes at the protein level.

More interestingly, the PPI network highlighted the antiviral proteins (AVPs) in psoriatic epidermis again, including Mx1, OAS2, and other interferon associated proteins (Figure 2A). As we know, AVPs in the skin, especially in the epidermis, play important roles in barrier defense (35, 36). Increased OAS2 expression in psoriasis has been reported in epigenome and transcriptome datasets (37, 38). Our proteomics data also showed OAS2 as a significant DEP in both psoriatic epidermis and serum of psoriatic individuals. Moreover, we found that OAS2 is also correlated with the severity of psoriasis determined by both PASI and BSA scores. Correlation analysis revealed OAS2 as a candidate epidermal biomarker reflecting psoriasis activity (Figures 3A–C). The expression level of OAS2 in the serum of psoriasis patients is dramatically higher than that of normal healthy controls. Remarkably, the expression of OAS2 in serum is highly positively correlated with the severity of psoriasis. The correlation coefficient is much higher than that of the epidermal tissue (Figures 3D–J). This evidence indicated that OAS2 is a novel sensitive biomarker for psoriasis activity. The transcriptional level of OAS2 in psoriatic skin is significantly reduced after anti-IL-17R treatment, time-dependently and dose-dependently (Figure 3L). OAS2 could be used as a biomarker to evaluate or monitor the efficacy of clinical treatment in the future.

It was clarified that inflamed keratinocytes were the main source of abnormally increased OAS2 in psoriasis skin (Figures 4A–D). *In vitro*, keratinocyte-derived OAS2 can be regulated by interferon beta, but not significantly changed by poly I:C. Interestingly, psoriasis-associated cytokines especially IL-17A and IL-6, can directly positively regulate keratinocytes to over-express OAS2 (Figures 4E–G). This data indicated that keratinocyte-derived OAS2 may be involved in the initiation of early psoriasis under the regulation of interferon, and may also participate in magnifying psoriatic epidermal inflammation with cytokines like IL-17A and IL-6.

*In vivo*, OAS2 specifically increased in IMQ-induced psoriasis-like mouse epidermis, which provided evidence for future studies using a mouse model (Figure 4H).

In conclusion, there are differences in protein expression profiles of psoriasis epidermis compared with healthy epidermis. Antibacterial peptides and antiviral proteins are two important functional protein clusters altering psoriasis innate immune status. OAS2 is a novel potential sensitive biomarker, which could predict the severity and activity of psoriasis, and it could also be used as an indicator to evaluate or predict the efficacy of clinical treatment.

## Data Availability Statement

Publicly available datasets were analyzed in this study. This data can be found here: Gene Expression Omnibus (GEO) (GSE53552).

## Ethics Statement

The studies involving human participants were reviewed and approved by Ethics committee of Second Affiliated Hospital, Zhejiang University School of Medicine. Written informed consent to participate in this study was provided by the participants' legal guardian/next of kin. The animal study was reviewed and approved by Ethics committee of Second Affiliated Hospital, Zhejiang University School of Medicine.

## Author Contributions

YZ, MZ, and X-YM: conceptualization. YZ, PW, and B-XY: data curation. YZ and X-YM: formal analysis and visualization. MZ and X-YM: funding acquisition. YZ, B-XY, X-YC, JZ, and X-XL: investigation. YZ, PW, and X-YM: methodology. X-YM and MZ: project administration and resources. X-YM: supervision. LL, Z-YW, JZ, and X-XL: validations. YZ and PW: writing—original draft preparation. YZ and X-YM: writing—review and editing. All authors contributed to the article and approved the submitted version.

## Conflict of Interest

The authors declare that the research was conducted in the absence of any commercial or financial relationships that could be construed as a potential conflict of interest.
