# Supplementary material for: Quantitative Proteomic Profile of Psoriatic Epidermis Identifies OAS2 as a Novel Biomarker for Disease Activity
Source: Front Immunol. 2020 Jul 31;11:1432. doi: 10.3389/fimmu.2020.01432 (PMC7410923; doi:10.3389/fimmu.2020.01432)
Supplement: Supplementary file 1 [file Data_Sheet_1.docx]

Supplementary Material

## Supplementary Figures


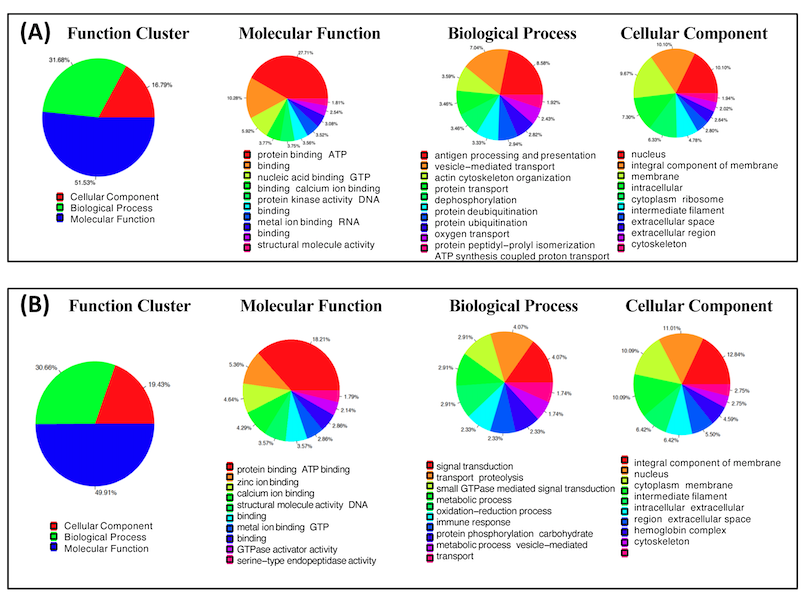


**Supplementary Figure 1.** Gene Ontology (GO) annotation of identified proteins and DEPs. (A,B) Functional clusters analysis according to GO, including molecular function(MF), biological process(BP) and cellular component (CC) for all identified proteins(A) and DEPs (B), respectively.


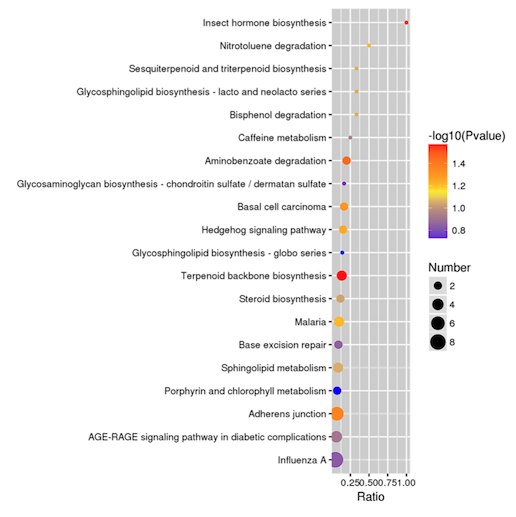


**Supplementary Figure 2.** Kyoto Encyclopedia of Genes and Genomes (KEGG) pathway analysis for DEPs


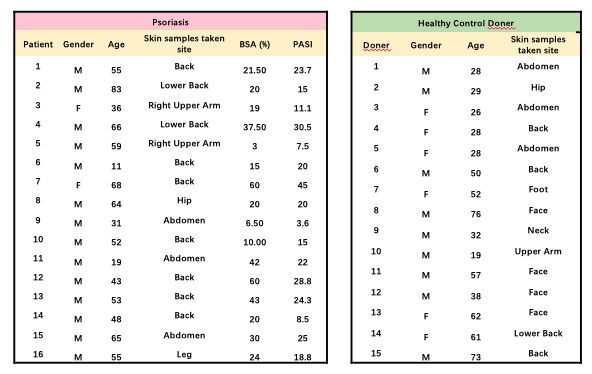


**Supplementary Table 1 and 2.** Demographics of the proteomic study cohort. Supplementary Table 1: Demographics of psoriasis patients; Supplementary Table 2: Demographics of healthy control

**Supplementary Table 3. The list of 269 DEPs.**

| **Protein ID** | **Description** | **Gene** | **Fold change (Pso/HC)** | ***P* vaule (Pso/HC)** |
| --- | --- | --- | --- | --- |
| Q2M2I5 | Keratin, type I cytoskeletal 24 | KRT24 | 0.115341239 | 0.03326267200 |
| Q15545 | Transcription initiation factor TFIID subunit 7 | TAF7 | 0.144100509 | 0.00000003010 |
| A0A1B0GUI1 | Ral GTPase-activating protein subunit alpha-1 | RALGAPA1 | 0.253627151 | 0.00053802000 |
| Q5JS54 | Proteasome assembly chaperone 4 | PSMG4 | 0.259119007 | 0.00000846000 |
| A0A024R2Z6 | Guanine nucleotide binding protein-like 3 (Nucleolar), isoform CRA_b | GNL3 | 0.289382848 | 0.00019655900 |
| Q9BSK0 | MARVEL domain-containing protein 1 | MARVELD1 | 0.298638728 | 0.00000256000 |
| Q9BTA9 | WW domain-containing adapter protein with coiled-coil | WAC | 0.304294705 | 0.00011811800 |
| Q03111 | Protein ENL | MLLT1 | 0.305919733 | 0.00000000277 |
| Q9BTY2 | Plasma alpha-L-fucosidase | FUCA2 | 0.307878982 | 0.00000024800 |
| Q86TV6 | Tetratricopeptide repeat protein 7B | TTC7B | 0.314153571 | 0.00000110000 |
| D3DTX6 | Neurabin-2 | PPP1R9B | 0.314210758 | 0.00000352000 |
| Q9H0V1 | Transmembrane protein 168 | TMEM168 | 0.333894582 | 0.00189214400 |
| Q9BST9 | Rhotekin | RTKN | 0.333996653 | 0.00063336000 |
| A0A0C4DG49 | Poliovirus receptor | PVR | 0.335624591 | 0.00001040000 |
| Q9H6F5 | Coiled-coil domain-containing protein 86 | CCDC86 | 0.33892293 | 0.00006370000 |
| Q9UQB3 | Catenin delta-2 | CTNND2 | 0.341282763 | 0.00014799600 |
| Q15700 | Disks large homolog 2 | DLG2 | 0.355257159 | 0.01299907300 |
| O60504 | Vinexin | SORBS3 | 0.358725879 | 0.00196956100 |
| Q86V87 | Protein FAM160B2 | FAM160B2 | 0.379976818 | 0.00075124700 |
| Q0MQR4 | Poly (ADP-ribose) glycohydrolase | PARG | 0.384926994 | 0.00001260000 |
| Q13485 | Mothers against decapentaplegic homolog 4 | SMAD4 | 0.396912732 | 0.00000937000 |
| Q9NYQ8 | Protocadherin Fat 2 | FAT2 | 0.399991941 | 0.00148270500 |
| Q8NEY1 | Neuron navigator 1 | NAV1 | 0.403116631 | 0.01633135300 |
| Q5W009 | RNA binding motif protein 17, isoform CRA_a | RBM17 | 0.403324881 | 0.00000109000 |
| Q8N1A6 | UPF0462 protein C4orf33 | C4orf33 | 0.409029816 | 0.00421218100 |
| P07204 | Thrombomodulin | THBD | 0.41422205 | 0.00000157000 |
| A0A024R0J7 | HCG1995540, isoform CRA_a | hCG_1995540 | 0.42645478 | 0.00012677000 |
| Q86TW2 | Uncharacterized aarF domain-containing protein kinase 1 | ADCK1 | 0.431961654 | 0.00001400000 |
| P05204 | Non-histone chromosomal protein HMG-17 | HMGN2 | 0.440509244 | 0.01767203900 |
| P42773 | Cyclin-dependent kinase 4 inhibitor C | CDKN2C | 0.443430877 | 0.00000032300 |
| A6NHB5 | Zinc finger MYM-type protein 3 | ZMYM3 | 0.446162114 | 0.00000001870 |
| Q5TZA2 | Rootletin | CROCC | 0.446448842 | 0.00001440000 |
| A0A024R6E8 | Galactosylceramidase, isoform CRA_c | GALC | 0.449944836 | 0.00000000777 |
| Q9Y5B8 | Nucleoside diphosphate kinase 7 | NME7 | 0.45089196 | 0.00000400000 |
| A0A223LX15 | Suppressor of fused | SUFU | 0.45591622 | 0.00362557700 |
| P15531 | Nucleoside diphosphate kinase A | NME1 | 0.457180167 | 0.00009400000 |
| B4DY17 | Methylthioribulose-1-phosphate dehydratase | APIP | 0.463851489 | 0.01414495500 |
| G5E9M7 | Transcription cofactor vestigial-like protein 4 | VGLL4 | 0.470606546 | 0.00000146000 |
| A6NKD9 | Coiled-coil domain-containing protein 85C | CCDC85C | 0.472379877 | 0.00155181500 |
| B3KR97 | cDNA FLJ33900 fis, clone CTONG2008262, highly similar to Low density lipoprotein receptor adapter protein 1 | | 0.475125882 | 0.00000038200 |
| Q9NYQ6 | Cadherin EGF LAG seven-pass G-type receptor 1 | CELSR1 | 0.482946554 | 0.00000004790 |
| A0A024QZI2 | FCGRT | hCG_1998059 | 0.486304429 | 0.00007180000 |
| P29372 | DNA-3-methyladenine glycosylase | MPG | 0.493911416 | 0.00830481700 |
| Q8WXD5 | Gem-associated protein 6 | GEMIN6 | 0.499485661 | 0.00017316300 |
| B4E368 | cDNA FLJ60058, highly similar to Myosin light chain 1, slow-twitch muscle A isoform | | 0.505117156 | 0.00000154000 |
| Q9BY89 | Uncharacterized protein KIAA1671 | KIAA1671 | 0.505405679 | 0.00236110200 |
| A0A087WT59 | Transthyretin | TTR | 0.505997122 | 0.00019377000 |
| A0A024R9B8 | Plasma glutamate carboxypeptidase, isoform CRA_a | PGCP | 0.510260337 | 0.00000208000 |
| A0A0I9N852 | MHC class II antigen | HLA-DQB1 | 0.512063465 | 0.02597360500 |
| A1L0S7 | TNS1 protein (Fragment) | TNS1 | 0.513333804 | 0.00032718600 |
| B4DX19 | cDNA FLJ57644, highly similar to Serum paraoxonase/arylesterase 1 (EC 3.1.1.2) | | 0.513434315 | 0.02767136600 |
| Q969S9 | Ribosome-releasing factor 2, mitochondrial | GFM2 | 0.513665405 | 0.00161558100 |
| I0B0K5 | Truncated profilaggrin | FLG | 0.515812269 | 0.00072158700 |
| P61244 | Protein max | MAX | 0.515972351 | 0.00002500000 |
| Q9Y3E0 | Vesicle transport protein GOT1B | GOLT1B | 0.516066516 | 0.00816619800 |
| B4DRW4 | cDNA FLJ59287, highly similar to Serine protease inhibitor Kazal-type 5 (Fragment) | | 0.516762517 | 0.00069350800 |
| Q8WU10 | Pyridine nucleotide-disulfide oxidoreductase domain-containing protein 1 | PYROXD1 | 0.517032792 | 0.04611413500 |
| V9HWK4 | Epididymis luminal protein 162 | HEL162 | 0.518203806 | 0.00000021700 |
| O43520 | Phospholipid-transporting ATPase IC | ATP8B1 | 0.518230391 | 0.00019437300 |
| Q69YL0 | Uncharacterized protein NCBP2-AS2 | NCBP2-AS2 | 0.520501984 | 0.00367920100 |
| E9PIE3 | Caveolae-associated protein 3 | CAVIN3 | 0.520600145 | 0.01847596400 |
| P35080 | Profilin-2 | PFN2 | 0.521376165 | 0.00331642000 |
| Q9NV56 | MRG/MORF4L-binding protein | MRGBP | 0.523105492 | 0.00299602100 |
| J3KQC6 | Transmembrane protease serine 13 | TMPRSS13 | 0.525027948 | 0.00007150000 |
| B2RB24 | cDNA, FLJ95266 |  | 0.527031351 | 0.00024311900 |
| Q96A08 | Histone H2B type 1-A | HIST1H2BA | 0.530304187 | 0.01915602700 |
| Q9UI09 | NADH dehydrogenase [ubiquinone] 1 alpha subcomplex subunit 12 | NDUFA12 | 0.531680146 | 0.00117428100 |
| Q7LFX5 | Carbohydrate sulfotransferase 15 | CHST15 | 0.532637076 | 0.03615774700 |
| B9EH95 | Armadillo repeat gene deletes in velocardiofacial syndrome | ARVCF | 0.540554263 | 0.00809199000 |
| B2RC94 | cDNA, FLJ95922 |  | 0.543611891 | 0.00001730000 |
| Q7Z4Q2 | HEAT repeat-containing protein 3 | HEATR3 | 0.545795084 | 0.00140942400 |
| A0A024R4Z1 | HCG2042749, isoform CRA_b | hCG_2042749 | 0.550388411 | 0.00004360000 |
| A0A0K2GN21 | BCKDHB protein | BCKDHB | 0.551586401 | 0.00380709300 |
| A6NL88 | Protein shisa-7 | SHISA7 | 0.552741263 | 0.04742727500 |
| A6XGP7 | Reticulon | NOGOC | 0.555328859 | 0.00165608000 |
| Q96RT7 | Gamma-tubulin complex component 6 | TUBGCP6 | 0.560868711 | 0.00049785600 |
| Q9NWY4 | Histone PARylation factor 1 | HPF1 | 0.561146689 | 0.00004530000 |
| Q75T13 | GPI inositol-deacylase | PGAP1 | 0.562811323 | 0.00199324700 |
| A0A024R0T1 | Short-chain dehydrogenase/reductase, isoform CRA_b | MGC4172 | 0.563396115 | 0.00001660000 |
| Q59ET9 | Mevalonate kinase (Fragment) |  | 0.563935446 | 0.00121523700 |
| Q9H0X9 | Oxysterol-binding protein-related protein 5 | OSBPL5 | 0.564381602 | 0.00017637400 |
| B5BU44 | CASP2 and RIPK1 domain containing adaptor with death domain | CRADD | 0.568277241 | 0.00007500000 |
| O94887 | FERM, RhoGEF and pleckstrin domain-containing protein 2 | FARP2 | 0.569163823 | 0.00114326700 |
| O14524 | Nuclear envelope integral membrane protein 1 | NEMP1 | 0.56956678 | 0.01167679600 |
| A8K0T6 | cDNA FLJ76736, highly similar to Homo sapiens sphingomyelin phosphodiesterase 3, neutral membrane (neutral sphingomyelinase II) (SMPD3), mRNA | | 0.570601564 | 0.02380569500 |
| Q9H6K4 | Optic atrophy 3 protein | OPA3 | 0.570861497 | 0.00000699000 |
| Q9NSB2 | Keratin, type II cuticular Hb4 | KRT84 | 0.571351499 | 0.04839156300 |
| Q5T200 | Zinc finger CCCH domain-containing protein 13 | ZC3H13 | 0.573556872 | 0.02682526800 |
| A0A1K0GXZ1 | Globin C1 | GLNC1 | 0.576425311 | 0.02057211300 |
| Q92752 | Tenascin-R | TNR | 0.577233007 | 0.02463760700 |
| P31025 | Lipocalin-1 | LCN1 | 0.577981925 | 0.00269540900 |
| B2RBM8 | cDNA, FLJ95596, highly similar to Homo sapiens activity-dependent neuroprotector (ADNP), mRNA | | 0.578947988 | 0.00064672000 |
| Q7RTP6 | [F-actin]-monooxygenase MICAL3 | MICAL3 | 0.579990892 | 0.00895086500 |
| B4E3D4 | cDNA FLJ56293, highly similar to Transmembrane glycoprotein NMB | | 0.580992657 | 0.00049786700 |
| Q96BW5 | Phosphotriesterase-related protein | PTER | 0.586259561 | 0.00017213600 |
| Q96T46 | Hemoglobin alpha 2 (Fragment) | HBA2 | 0.586564227 | 0.01914620300 |
| P19012 | Keratin, type I cytoskeletal 15 | KRT15 | 0.588253078 | 0.00802055700 |
| Q494U1 | Pleckstrin homology domain-containing family N member 1 | PLEKHN1 | 0.588656039 | 0.00028509700 |
| J3KQR7 | Centrosomal protein of 170 kDa protein B | CEP170B | 0.589032744 | 0.00064739600 |
| A0A024R8Y2 | POU domain protein | POU2F1 | 0.594024627 | 0.03955634700 |
| Q969M7 | NEDD8-conjugating enzyme UBE2F | UBE2F | 0.598632275 | 0.00065027000 |
| A0A024RB84 | V-erb-b2 erythroblastic leukemia viral oncogene homolog 3 (Avian), isoform CRA_a | ERBB3 | 0.598993795 | 0.00001340000 |
| Q96N95 | Zinc finger protein 396 | ZNF396 | 0.599148367 | 0.00366947500 |
| D3DTX7 | Collagen, type I, alpha 1, isoform CRA_a | COL1A1 | 0.600245414 | 0.00158859600 |
| P81605 | Dermcidin | DCD | 0.600521848 | 0.02046683200 |
| P68431 | Histone H3.1 | HIST1H3A | 0.601727231 | 0.00068832000 |
| Q7L3T8 | Probable proline--tRNA ligase, mitochondrial | PARS2 | 0.602061112 | 0.00066851900 |
| Q9UJC5 | SH3 domain-binding glutamic acid-rich-like protein 2 | SH3BGRL2 | 0.602184627 | 0.00004750000 |
| P22415 | Upstream stimulatory factor 1 | USF1 | 0.602698608 | 0.00071592500 |
| Q96ME1 | F-box/LRR-repeat protein 18 | FBXL18 | 0.60285629 | 0.00101215500 |
| A0A024RDT3 | Calcium binding protein 39-like, isoform CRA_a | CAB39L | 0.60314133 | 0.01772136400 |
| A0A087X266 | Transmembrane protein 120A | TMEM120A | 0.604908437 | 0.00019622100 |
| Q86SQ8 | Beta-defensin-1 (Fragment) | HBD1 | 0.604913798 | 0.00000019700 |
| O43312 | Metastasis suppressor protein 1 | MTSS1 | 0.609741724 | 0.00008040000 |
| O60245 | Protocadherin-7 | PCDH7 | 0.60986708 | 0.00017495800 |
| A8K4L6 | Vang-like protein |  | 0.609933559 | 0.00334542400 |
| Q9NR33 | DNA polymerase epsilon subunit 4 | POLE4 | 0.611986926 | 0.02371915400 |
| Q9BWH6 | RNA polymerase II-associated protein 1 | RPAP1 | 0.612434709 | 0.00242997200 |
| Q96RN5 | Mediator of RNA polymerase II transcription subunit 15 | MED15 | 0.61258271 | 0.01457844700 |
| B4DP06 | cDNA FLJ57133, highly similar to Bifunctional purine biosynthesis protein PURH | | 0.617421431 | 0.01933341700 |
| P07205 | Phosphoglycerate kinase 2 | PGK2 | 0.620916844 | 0.00824663200 |
| Q9NP61 | ADP-ribosylation factor GTPase-activating protein 3 | ARFGAP3 | 0.621160757 | 0.00000721000 |
| Q9BY12 | S phase cyclin A-associated protein in the endoplasmic reticulum | SCAPER | 0.621673252 | 0.00154843300 |
| Q658Y4 | Protein FAM91A1 | FAM91A1 | 0.622246066 | 0.00202790100 |
| Q8WZ64 | Arf-GAP with Rho-GAP domain, ANK repeat and PH domain-containing protein 2 | ARAP2 | 0.62332886 | 0.00340826300 |
| P21359 | Neurofibromin | NF1 | 0.62424244 | 0.00411337400 |
| Q96GL3 | IRF3 protein | IRF3 | 0.624593073 | 0.01614666000 |
| Q6NSJ5 | Volume-regulated anion channel subunit LRRC8E | LRRC8E | 0.627197713 | 0.00208822100 |
| A0A024R926 | Chromosome 1 open reading frame 21, isoform CRA_a | C1orf21 | 0.627590368 | 0.00584788900 |
| B2RB94 | cDNA, FLJ95380, highly similar to Homo sapiens vacuolar protein sorting 41 (yeast) (VPS41), transcript variant 1, mRNA | | 0.6282495 | 0.03732233500 |
| A0A024RDS2 | Periostin, osteoblast specific factor, isoform CRA_c | POSTN | 0.630336939 | 0.00161715300 |
| B4E1Z4 | cDNA FLJ55673, highly similar to Complement factor B (EC 3.4.21.47) | | 0.630395597 | 0.00149884600 |
| A8K701 | cDNA FLJ77574, highly similar to Homo sapiens iduronidase, alpha-L- (IDUA), mRNA | | 0.631160932 | 0.01370578400 |
| F5H5R8 | Arylamine N-acetyltransferase 1 | NAT1 | 0.631677855 | 0.00020583700 |
| Q17R23 | Stonin 2 | STON2 | 0.632527479 | 0.03843468700 |
| B2R533 | cDNA, FLJ92320, highly similar to Homo sapiens glutathione S-transferase theta 2 (GSTT2), mRNA | | 0.634187584 | 0.00151684500 |
| Q2NL82 | Pre-rRNA-processing protein TSR1 homolog | TSR1 | 0.634333516 | 0.00197175400 |
| E9LUH4 | Methyl-CpG-binding protein 2 | MECP2 | 0.635440698 | 0.01638520000 |
| Q6NUJ1 | Proactivator polypeptide-like 1 | PSAPL1 | 0.635549717 | 0.00553787700 |
| O75382 | Tripartite motif-containing protein 3 | TRIM3 | 0.635727699 | 0.00594391200 |
| Q6P9B9 | Integrator complex subunit 5 | INTS5 | 0.636987824 | 0.00285574500 |
| Q96P48 | Arf-GAP with Rho-GAP domain, ANK repeat and PH domain-containing protein 1 | ARAP1 | 0.637881399 | 0.00011265400 |
| B4DKM0 | cDNA FLJ51883, highly similar to Mitochondrial 39S ribosomal protein L3 | | 0.638408374 | 0.01028404900 |
| A8K4I8 | cDNA FLJ78131, highly similar to Homo sapiens nipsnap homolog 1 (C. elegans) (NIPSNAP1), mRNA | | 0.641095729 | 0.00234116500 |
| Q8NHP6 | Motile sperm domain-containing protein 2 | MOSPD2 | 0.642873873 | 0.01618181400 |
| Q7Z3Z0 | Keratin, type I cytoskeletal 25 | KRT25 | 0.644109634 | 0.01401810700 |
| Q9BQP7 | Mitochondrial genome maintenance exonuclease 1 | MGME1 | 0.644508337 | 0.00147150600 |
| A8K9X0 | Protein YIPF |  | 0.645441354 | 0.00066491600 |
| A0A024R6D1 | NIMA (Never in mitosis gene a)-related kinase 9, isoform CRA_a | NEK9 | 0.645750023 | 0.00006610000 |
| Q9HCC8 | Glycerophosphoinositol inositolphosphodiesterase GDPD2 | GDPD2 | 0.646018933 | 0.01280223900 |
| Q6IEH8 | Nipped-B protein | NIPBL | 0.647402654 | 0.01083248700 |
| Q5D862 | Filaggrin-2 | FLG2 | 0.648586513 | 0.03875871300 |
| Q9BT40 | Inositol polyphosphate 5-phosphatase K | INPP5K | 0.650325973 | 0.00009350000 |
| Q9Y2H0 | Disks large-associated protein 4 | DLGAP4 | 0.650394098 | 0.00509914000 |
| P48067 | Sodium- and chloride-dependent glycine transporter 1 | SLC6A9 | 0.651066277 | 0.01056429700 |
| Q14657 | EKC/KEOPS complex subunit LAGE3 | LAGE3 | 0.651895412 | 0.00732014000 |
| O94913 | Pre-mRNA cleavage complex 2 protein Pcf11 | PCF11 | 0.653000881 | 0.00137505200 |
| A0A0A0MSZ4 | NADPH:adrenodoxin oxidoreductase, mitochondrial | FDXR | 0.65336043 | 0.00002020000 |
| Q13445 | Transmembrane emp24 domain-containing protein 1 | TMED1 | 0.653464131 | 0.00106319000 |
| B3KM74 | cDNA FLJ10425 fis, clone NT2RP1000326, highly similar to Metaxin-2 | | 0.654197607 | 0.01293011400 |
| Q6UXB8 | Peptidase inhibitor 16 | PI16 | 0.65465509 | 0.00619256400 |
| B7Z6H5 | cDNA FLJ55938, highly similar to Dedicator of cytokinesis protein 9 | | 0.654763683 | 0.00062887200 |
| B2RE11 | cDNA, FLJ96865 |  | 0.656749175 | 0.00006150000 |
| A0A024R1X5 | Beclin 1 (Coiled-coil, myosin-like BCL2 interacting protein), isoform CRA_b | BECN1 | 0.65738771 | 0.00006300000 |
| P62745 | Rho-related GTP-binding protein RhoB | RHOB | 0.660853531 | 0.04451639300 |
| Q9UPP1 | Histone lysine demethylase PHF8 | PHF8 | 0.661845125 | 0.01216252800 |
| B2R6J3 | cDNA, FLJ92974, highly similar to Homo sapiens methylmalonic aciduria (cobalamin deficiency) type B(MMAB), mRNA | | 0.662001203 | 0.00139469100 |
| A0A087X1U0 | Tryptase beta-2 | TPSB2 | 0.662698038 | 0.04585841100 |
| Q6ZNJ1 | Neurobeachin-like protein 2 | NBEAL2 | 0.663857346 | 0.00512496000 |
| L7RRS0 | Phosphatidylinositol-4-phosphate 3-kinase, catalytic subunit type 2 alpha | PIK3C2A | 0.665237216 | 0.00565124700 |
| A1L4H1 | Soluble scavenger receptor cysteine-rich domain-containing protein SSC5D | SSC5D | 0.666152545 | 0.00259850300 |
| P09758 | Tumor-associated calcium signal transducer 2 | TACSTD2 | 0.666473668 | 0.00024819600 |
| A0A024R0X0 | AP1 gamma subunit binding protein 1, isoform CRA_d | AP1GBP1 | 0.666655289 | 0.00370168500 |
| O00755 | Protein Wnt-7a | WNT7A | 3.847494846 | 0.00001260000 |
| Q7Z7N9 | Transmembrane protein 179B | TMEM179B | 3.723506051 | 0.00000350000 |
| Q86SG5 | Protein S100-A7A | S100A7A | 3.545246343 | 0.00002090000 |
| P06702 | Protein S100-A9 | S100A9 | 3.485090794 | 0.02372586600 |
| Q9Y3A6 | Transmembrane emp24 domain-containing protein 5 | TMED5 | 3.467998851 | 0.00006150000 |
| Q9NVJ2 | ADP-ribosylation factor-like protein 8B | ARL8B | 2.825262969 | 0.00001560000 |
| Q5U7J2 | Beta-defensin 3 (Fragment) | DEFB103A | 2.542675982 | 0.00001400000 |
| P48594 | Serpin B4 | SERPINB4 | 2.41391581 | 0.00054016500 |
| P29508 | Serpin B3 | SERPINB3 | 2.300585581 | 0.00023540800 |
| K7ENW6 | Keratin, type I cytoskeletal 16 (Fragment) | KRT16 | 2.277768911 | 0.04416345800 |
| P19957 | Elafin | PI3 | 2.260225878 | 0.00017405100 |
| H0YDF6 | NHS-like protein 1 (Fragment) | NHSL1 | 2.191082686 | 0.00010886900 |
| Q5QPA5 | 39S ribosomal protein S18a, mitochondrial (Fragment) | MRPS18A | 2.159497413 | 0.00079206900 |
| P31151 | Protein S100-A7 | S100A7 | 2.087368329 | 0.00584507800 |
| Q9BYN8 | 28S ribosomal protein S26, mitochondrial | MRPS26 | 2.080820048 | 0.00000519000 |
| A0A075B6R9 | Immunoglobulin kappa variable 2D-24 (non-functional) (Fragment) | IGKV2D-24 | 2.072378579 | 0.00109807300 |
| I3VM53 | F-box and leucine-rich repeat protein 11, isoform CRA_a | KDM2A | 2.035174281 | 0.00002700000 |
| A0A024R1C1 | Peptidylprolyl isomerase (Cyclophilin)-like 2, isoform CRA_b | PPIL2 | 2.030948135 | 0.00045802800 |
| Q9H1A3 | Methyltransferase-like protein 9 | METTL9 | 1.997460292 | 0.03058221300 |
| A0A125QYY5 | GCT-A9 light chain variable region (Fragment) |  | 1.976130682 | 0.00850739600 |
| A0A024QYX0 | Emopamil binding protein | EBP | 1.943404878 | 0.00360894100 |
| Q5T765 | Interferon-induced protein with tetratricopeptide repeats 3, isoform CRA_a | IFIT3 | 1.912811311 | 0.00000398000 |
| P29373 | Cellular retinoic acid-binding protein 2 | CRABP2 | 1.891725132 | 0.00051298600 |
| L0R819 | Alternative protein ASNSD1 | ASNSD1 | 1.887880406 | 0.00757019100 |
| A0A0A0MQX1 | Unconventional myosin-X | MYO10 | 1.857125066 | 0.03572034300 |
| B2R6A9 | cDNA, FLJ92868, highly similar to Homo sapiens HIRA interacting protein 3 (HIRIP3), mRNA | | 1.853678284 | 0.00003780000 |
| O75298 | Reticulon-2 | RTN2 | 1.850649551 | 0.02394338700 |
| Q9UKY7 | Protein CDV3 homolog | CDV3 | 1.838721982 | 0.01280086900 |
| A8K7T0 | Kynureninase | KYNU | 1.824291689 | 0.00002670000 |
| A0A087X0V5 | 2-5-oligoadenylate synthase 2 | OAS2 | 1.823237843 | 0.00001810000 |
| Q8WU76 | Sec1 family domain-containing protein 2 | SCFD2 | 1.815284157 | 0.00126886000 |
| Q9UKQ9 | Kallikrein-9 | KLK9 | 1.80447757 | 0.00390496200 |
| Q9ULI0 | ATPase family AAA domain-containing protein 2B | ATAD2B | 1.780046414 | 0.00003700000 |
| Q8IWR0 | Zinc finger CCCH domain-containing protein 7A | ZC3H7A | 1.771068448 | 0.00000419000 |
| Q6MZM0 | Hephaestin-like protein 1 | HEPHL1 | 1.761108078 | 0.00001060000 |
| P05109 | Protein S100-A8 | S100A8 | 1.75504946 | 0.00527256700 |
| B4DS41 | cDNA FLJ55173, highly similar to Nuclear distribution protein nudE-like 1 | | 1.739427232 | 0.00000193000 |
| Q5U0I6 | H.sapiens ras-related Hrab1A protein | RAB1A | 1.734159279 | 0.00018868800 |
| P30260 | Cell division cycle protein 27 homolog | CDC27 | 1.730149606 | 0.00137271300 |
| O14933 | Ubiquitin/ISG15-conjugating enzyme E2 L6 | UBE2L6 | 1.727264772 | 0.00111375200 |
| P78362 | SRSF protein kinase 2 | SRPK2 | 1.724174338 | 0.00171911800 |
| Q8IZ81 | ELMO domain-containing protein 2 | ELMOD2 | 1.719945908 | 0.00037927100 |
| Q9H814 | Phosphorylated adapter RNA export protein | PHAX | 1.713058655 | 0.00012562700 |
| C9K0J5 | Ras association (RalGDS/AF-6) and pleckstrin homology domains 1, isoform CRA_b | RAPH1 | 1.710043603 | 0.00393421400 |
| X6R8F3 | Neutrophil gelatinase-associated lipocalin | LCN2 | 1.706051915 | 0.00613259400 |
| P42695 | Condensin-2 complex subunit D3 | NCAPD3 | 1.704518027 | 0.00093156100 |
| A0A024R518 | Interleukin-1 | IL1F5 | 1.693103646 | 0.00055184900 |
| Q01469 | Fatty acid-binding protein, epidermal | FABP5 | 1.685854013 | 0.00048525300 |
| Q4TZM4 | Hemoglobin beta chain (Fragment) | HBB | 1.674257458 | 0.02651287800 |
| P20591 | Interferon-induced GTP-binding protein Mx1 | MX1 | 1.663041523 | 0.00011528100 |
| B2R914 | Gap junction protein |  | 1.651962889 | 0.00173609500 |
| A8K4D5 | Kynureninase | KYNU | 1.647414593 | 0.00555612000 |
| A0A0S2Z428 | HCG2039812, isoform CRA_b (Fragment) | KRT6A | 1.64475672 | 0.00057614300 |
| P22760 | Arylacetamide deacetylase | AADAC | 1.641243299 | 0.00019931000 |
| B2R6A5 | cDNA, FLJ92862, highly similar to Homo sapiens solute carrier family 16 (monocarboxylic acid transporters), member 1 (SLC16A1), mRNA | | 1.638740736 | 0.02918861100 |
| Q7L5D6 | Golgi to ER traffic protein 4 homolog | GET4 | 1.638389238 | 0.00068252700 |
| O14519 | Cyclin-dependent kinase 2-associated protein 1 | CDK2AP1 | 1.635814654 | 0.00923362800 |
| B0AZP3 | cDNA, FLJ79483, highly similar to Homo sapiens guanylate binding protein family, member 6 (GBP6), mRNA | | 1.613752737 | 0.00160018000 |
| O60248 | Protein SOX-15 | SOX15 | 1.613497603 | 0.00001090000 |
| I3NI25 | LisH domain-containing protein FOPNL | FOPNL | 1.6115454 | 0.03316652100 |
| Q8TF71 | Monocarboxylate transporter 10 | SLC16A10 | 1.603123794 | 0.04818059200 |
| A0A087WZV0 | Inositol hexakisphosphate and diphosphoinositol-pentakisphosphate kinase 2 | PPIP5K2 | 1.592215349 | 0.01077322000 |
| B3VMW0 | Lactoferrin |  | 1.586665893 | 0.01722695400 |
| Q12830 | Nucleosome-remodeling factor subunit BPTF | BPTF | 1.586294564 | 0.00007430000 |
| Q96JK2 | DDB1- and CUL4-associated factor 5 | DCAF5 | 1.582498926 | 0.00015745700 |
| Q9Y2I8 | WD repeat-containing protein 37 | WDR37 | 1.579297246 | 0.00123021500 |
| P29034 | Protein S100-A2 | S100A2 | 1.572962486 | 0.00362158300 |
| Q03164 | Histone-lysine N-methyltransferase 2A | KMT2A | 1.570684008 | 0.00865164700 |
| A8K7D9 | Importin subunit alpha |  | 1.567074408 | 0.00000002830 |
| Q6NUL7 | SPTLC1 protein | SPTLC1 | 1.565735806 | 0.00858600700 |
| A8K6I5 | Signal transducer and activator of transcription |  | 1.56563568 | 0.00563922200 |
| Q9UEK9 | Keratin (Fragment) | KRT5 | 1.560788528 | 0.04789069100 |
| P04259 | Keratin, type II cytoskeletal 6B | KRT6B | 1.560444531 | 0.00112305100 |
| A8K5A6 | cDNA FLJ76827, highly similar to Homo sapiens BUB1 budding uninhibited by benzimidazoles 1 homolog (yeast) (BUB1), mRNA | | 1.552340121 | 0.03748642500 |
| A0A1R3UCE8 | Kallikrein I | KLNI | 1.549751289 | 0.02032462900 |
| Q8IY21 | Probable ATP-dependent RNA helicase DDX60 | DDX60 | 1.548235474 | 0.00001080000 |
| Q6ZNF0 | Acid phosphatase type 7 | ACP7 | 1.545178641 | 0.00563248200 |
| A8KA05 | Protein argonaute-3 | EIF2C3 | 1.545043799 | 0.00430182700 |
| Q13427 | Peptidyl-prolyl cis-trans isomerase G | PPIG | 1.543672665 | 0.00069777300 |
| Q9NZH8 | Interleukin-36 gamma | IL36G | 1.541039529 | 0.00018167700 |
| Q5HYI4 | Uncharacterized protein DKFZp686B0215 | DKFZp686B0215 | 1.53820316 | 0.02505885400 |
| Q9BV10 | Dol-P-Man:Man(7)GlcNAc(2)-PP-Dol alpha-1,6-mannosyltransferase | ALG12 | 1.537522412 | 0.00000047100 |
| A0A087X0R6 | Sorting nexin-12 | SNX12 | 1.536677672 | 0.00289311400 |
| O75452 | Retinol dehydrogenase 16 | RDH16 | 1.530232956 | 0.00013548300 |
| A8KA76 | cDNA FLJ78660, highly similar to Homo sapiens interferon induced with helicase C domain 1 (IFIH1), mRNA | | 1.529133657 | 0.00265276500 |
| B4DHD2 | cDNA FLJ55458, highly similar to Programmed cell death 6-interacting protein | | 1.525848297 | 0.01371952900 |
| B2RDU6 | Mevalonate kinase | MVK | 1.524332252 | 0.00007420000 |
| B3KUB6 | cDNA FLJ39529 fis, clone PUAEN2004067, highly similar to Band 4.1-like protein 1 | | 1.521621588 | 0.03056412600 |
| A0A024R912 | Uridine-cytidine kinase | UCK2 | 1.520295148 | 0.00020020300 |
| Q86Y56 | Dynein assembly factor 5, axonemal | DNAAF5 | 1.513815283 | 0.00276132400 |
| P28288 | ATP-binding cassette sub-family D member 3 | ABCD3 | 1.512501853 | 0.00945155300 |
| Q6FI30 | Nuclear factor 1 | NFIC | 1.510100188 | 0.04558225100 |
| Q8TDZ2 | [F-actin]-monooxygenase MICAL1 | MICAL1 | 1.509571496 | 0.02148891500 |
| A0A024R1I5 | SEC14-like 2 (S. cerevisiae), isoform CRA_b | SEC14L2 | 1.504151501 | 0.03264781700 |
| Q96EM0 | Trans-3-hydroxy-L-proline dehydratase | L3HYPDH | 1.503214248 | 0.00046147900 |
| B4E2S3 | cDNA FLJ56561 |  | 1.502281304 | 0.00086782500 |

**Supplementary Table 3.** The list of 269 DEPs in psoriatic epidemis
